# Autocrine IGF-II-Associated Cancers: From a Rare Paraneoplastic Event to a Hallmark in Malignancy

**DOI:** 10.3390/biomedicines12010040

**Published:** 2023-12-22

**Authors:** Pierluigi Scalia, Ignazio R. Marino, Salvatore Asero, Giuseppe Pandini, Adda Grimberg, Wafik S. El-Deiry, Stephen J. Williams

**Affiliations:** 1 The ISOPROG-Somatolink EPFP Research Network, Philadelphia, PA 19102, USA; 93100 Caltanissetta, Italy; 2 Department of Surgery, Sidney Kimmel Medical College, Thomas Jefferson University, Philadelphia, PA 19107, USA; 3 ARNAS Garibaldi, UOC Chirurgia Oncologica, Nesima, 95122 Catania, Italy; 4 Perelman School of Medicine, University of Pennsylvania, Children’s Hospital of Philadelphia, Philadelphia, PA 19104, USA; 5 Legorreta Cancer Center, Brown University, Providence, RI 02903, USA; 6 Department of Biology, College of Science and Technology, Temple University, Philadelphia, PA 19122, USA

**Keywords:** NICTH, EPTH, NSILA, IGF1-IGF2 (gene), IGF-I-IGF-II (protein), IR^A^, IGF-IR, HR^A/B^, IGF2oma, IGF2ST, SpI2-6/IGF-IIR

## Abstract

The paraneoplastic syndrome referred in the literature as non-islet-cell tumor hypoglycemia (NICTH) and extra-pancreatic tumor hypoglycemia (EPTH) was first reported almost a century ago, and the role of cancer-secreted IGF-II in causing this blood glucose-lowering condition has been widely established. The landscape emerging in the last few decades, based on molecular and cellular findings, supports a broader role for IGF-II in cancer biology beyond its involvement in the paraneoplastic syndrome. In particular, a few key findings are constantly observed during tumorigenesis, (a) a relative and absolute increase in fetal insulin receptor isoform (IR^A^) content, with (b) an increase in IGF-II high-molecular weight cancer-variants (big-IGF-II), and (c) a stage-progressive increase in the IGF-II autocrine signal in the cancer cell, mostly during the transition from benign to malignant growth. An increasing and still under-exploited combinatorial pattern of the IGF-II signal in cancer is shaping up in the literature with respect to its transducing receptorial system and effector intracellular network. Interestingly, while surgical and clinical reports have traditionally restricted IGF-II secretion to a small number of solid malignancies displaying paraneoplastic hypoglycemia, a retrospective literature analysis, along with publicly available expression data from patient-derived cancer cell lines conveyed in the present perspective, clearly suggests that IGF-II expression in cancer is a much more common event, especially in overt malignancy. These findings strengthen the view that (1) IGF-II expression/secretion in solid tumor-derived cancer cell lines and tissues is a broader and more common event compared to the reported IGF-II association to paraneoplastic hypoglycemia, and (2) IGF-II associates to the commonly observed autocrine loops in cancer cells while IGF-I cancer-promoting effects may be linked to its paracrine effects in the tumor microenvironment. Based on these evidence-centered considerations, making the autocrine IGF-II loop a hallmark for malignant cancer growth, we here propose the functional name of IGF-II secreting tumors (IGF-IIsT) to overcome the view that IGF-II secretion and pro-tumorigenic actions affect only a clinical sub-group of rare tumors with associated hypoglycemic symptoms. The proposed scenario provides an updated logical frame towards biologically sound therapeutic strategies and personalized therapeutic interventions for currently unaccounted IGF-II-producing cancers.

## 1. Introduction

**Figure 1:**
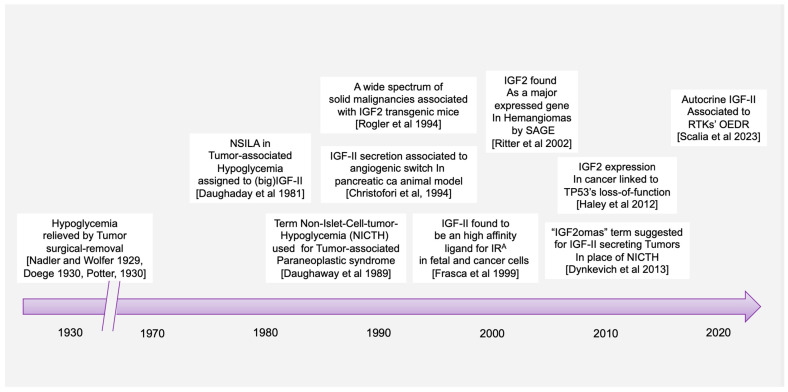
Historical timeline for key discoveries conferring a central role to IGF-II in the Insulin/IGF receptorial system in cancer. References within figure are as follows: Nadler and Wolfer, 1929 [2]; Doege 1930 [3]; Potter 1930 [4]; Daughaday et al., 1981 [13]; Daughaday 1989 [6]; Rogler et al., 1994; [14]; Christifori et al., 1994 [15]; Frasca et al., 1999 [16]; Ritter et al., 2002 [17]; Haley et al., 2012 [18]; Dynkevich et al., 2013 [5]; Salia et al., 2023 [19].

The earliest reports of the paraneoplastic syndrome associating what has been later referred as non-suppressible insulin-like activity (NSILA) [1] to hypoglycemia in cancer goes back to reports from W.H. Nadler and J.A. Wolfer in 1929 [2] and Karl W. Doege [3] and R.P. Potter in 1930 [4]. In possible oversight of the earlier report, the term of Doege–Potter Syndrome was adopted to describe these surgically treated intrathoracic tumors associated with hypoglycemia. Later reports confirmed that paraneoplastic hypoglycemia could indeed be found in cancers from all other (extra-thoracic) body districts and not limited to those of fibrous (connective/soft tissue) origin (namely sarcomas), as already suggested by the first under-looked report in 1929, but almost equally associated with epithelial/parenchymal tissue-derived cancers (carcinomas) [5]. The first findings linking IGF-II to cancer paraneoplastic hypoglycemia were related in the work of Doughaday et al. [6,7]. The added value of his work is linked to the observation that cancer-secreted IGF-II differs from physiologically produced IGF-II and that such difference confers cancer-secreted (“Big”)IGF-II key biologic advantages underlying its now widely proven autocrine loop effects. Specifically, cancer-secreted IGF-II corresponds to the IGF-II pro-hormone retaining its E domain, allowing its O-Glycosylation [6,8,9]. This processing defect increases the life-span and bioavailability of the IGF-II variants, both in the tumor microenvironment and in the systemic circulation, by reducing binding to IGFBP-3 and the IGF-II scavenger protein SpI2-6 (deceivingly known as the IGF-II “receptor” but actually causing IGF-II sequestration and degradation) [10,11,12]. The timeline of key discoveries connecting IGF-II to paraneoplastic hypoglycemia and proving its unique biological features are conveyed in Figure 1.

## 2. Cancer-Secreted IGF-II and Paraneoplastic Hypoglycemia: Is There Sufficient Evidence Supporting IGF-II as the Key IGF Ligand Involved in Solid Malignancy?

Despite the finding of IGF-II expression and secretion in cancer having long being established through the literature (Table 1), some authors have been supporting a comparable/interchangeable cancer-driving role for IGF-I, which mediates growth hormone effects during post-natal development in all vertebrates. This view, which implies a biological equivalence for IGF-I and IGF-II in cancer, cannot be supported any longer based on a number of available lines of evidence further discussed herein. Among these, we are adding the literature and expression correlation analysis assigning cancer-secreted IGF-II a distinctive hallmark compared to IGF-I. This is summarized in Figure 1 and Table 1 and discussed herein.

Specifically, through the present retrospective analysis of the reported cases of cancer-associated hypoglycemia (Figure 1 and Table 1), we found that secreted-IGF-II constitutes the absolute majority (95%, 171 out of 180) of the associated IGF. We also found IGF-II to be linked to a wide number of solid cancers in patients with these paraneoplastic symptoms, independently of the cancer embryological origin (spanning from sarcomas and carcinomas). On the other hand, we found only 18 reported cases of IGF-I-secreting cancers, out of which there were nine cases where IGF-II was not measured, three cases relating to patients diagnosed with Acromegaly and IGF-I levels normalized after treatment, two cases associated with patients diagnosed with childhood Leukemia but with initial levels of IGF-I and IGF-II that were below normal range and which both further decreased after treatment and remission, two cases describing patients with a benign pituitary tumor, and two cases reporting patients diagnosed with Medulloblastoma and with IGF-I levels that normalized after surgery with no significant changes in IGF-II levels.

Overall, the number of cancers with hypoglycemic symptoms secreting IGF-II and associated with malignancies exceed the number and types of tumors (mainly pituitary in origin) linked to IGF-I expression/secretion. This is in apparent conflict with the epidemiology results displaying an association between IGF-I blood levels and solid cancer risk.

In this context, it is useful to trace back the lines of evidence which have led us to the view linking IGF-I and IGF-II to cancer in order to highlight eventual incongruences. A literature search again provides a quantity of actionable evidence to this regard. In particular, given the physiological roles of these growth factors on developmental growth such as those summarized based on genetic knock-down work in rodents [20,21,22,23,24], we specifically minimized the inclusion of studies on IGFs genetics and physiology and focused our literature review on the work on IGFs in solid malignancies at the cellular, molecular, and clinical levels.

This parallel search shows that only a minor number of published works have looked at both IGF-I and IGF-II in the same studied cancer model (cellular, molecular, clinical, or epidemiologic). On the other hand, a larger number of highly referenced studies (e.g., trying to reconstitute signaling events) in vitro have made extensive use of exogenous stimulation of IGF-IR-expressing cellular models, often using supraphysiologic amounts of IGF-I (e.g., 100 nM and higher) without properly integrating or reconstituting the in vivo ligands and receptors landscape in their experimental design.

Overall, such a reductionistic in vitro approach, if it has, on one hand, advanced our understanding on the mechanistic aspects of this ligands/receptor system, has, on the other hand, been misleading in that the following aspects:

(a) It does not take in consideration the actual in vivo IGFs ligands and receptors co-expression context, which, taken together, supports a specific and independent role for cancer-secreted IGF-II and its autocrine loops;
(b) It does not succeed in explaining the failure of the individual pharmacological blockers of IGF-IR in clinical trials towards meeting the invoked therapeutic advantages suggested by the in vitro and epidemiologic studies;
(c) It has kept excluding alternative hypotheses and proper controls in experimental design which have been suggested by additional evidence available since the late nineties and proving the existence of an IGF-II- Insulin fetal receptor isoform (IR^A^) axis in mammalian fetal and cancer cells [16], as well as the expression and biological impact of IGF-IR/IR isoform-specific hybrids [25] in the studied cancer models.

Arguably, even relatively recent studies published on reputable journals [26] keep restricting the study focus on the IGF-I/IGF-IR axis as a standalone system in cancer without including parallel analysis of the IGF-II/IR^A^ ligand/RTK system in their experimental design [27], reiterating the persistence of an unsupported bias in the interpretation of the available experimental and observational data. Our retroactive analysis of the published literature in regard to the IGFs’ involvement in cancer cases displaying NSILA-dependent hypoglycemia is conveyed in Table 1 and graphically summarized in Figure 1 above.

Table 1: Based on the available literature out of all cases of cancer-associated hypoglycemia (1949 cases since 1929), 171 cases (10.3%) were reported after the available immunometric test had been developed and could be clearly associated with high IGF-II secretion levels versus 38 cases also reporting increased levels of IGF-I (1.94%) along with IGF-II. IGF-II association with such paraneoplastic condition was underestimated due to the fact that the IGF-II testing had been made available only in the early 1970′s. * Compatible with cancer stromal component secretion as source of increased levels.

## 3. IGF-II Over-Expression Is a Common Event in Cancer Cell-Lines

**Figure 2:**
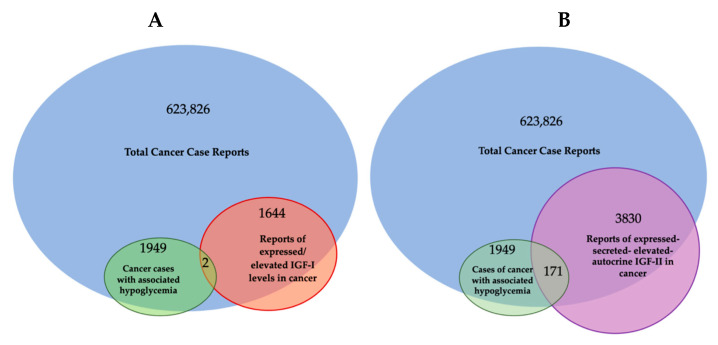
Cancer cases in the scientific literature exhibiting hypoglycemia associated with IGFs secretion. Venn diagram produced with online software available at https://www.meta-chart.com/venn#/display (accessed on 21 October 2023). (**A**) IGF-I-related cases. (**B**) IGF-II-related cases. The data analysis was the result of a PubMed literature search conveyed in Table 1. Note: the IGF reports in the Venn diagram followed the advent of IGF-I and IGF-II immunometric testing development (1972).

While IGF2 expression in somatic cells is regulated via parental imprinting, its regulation in cancer cells is determined by a combination of both imprinting and transcriptional regulation mechanisms reviewed elsewhere [[28], ibidem]. Ultimately, independently of the underlying genetic, translational, and post-translational mechanisms involved, the phenotypic and functional effects of such increased expression is reflected in the secretion of high molecular IGF-II pro-hormone variants [9] and its autocrine signal, which has been associated with both paraneoplastic hypoglycemia and malignancy (summarized in Figure 2 and Table 1).

**Figure 3:**
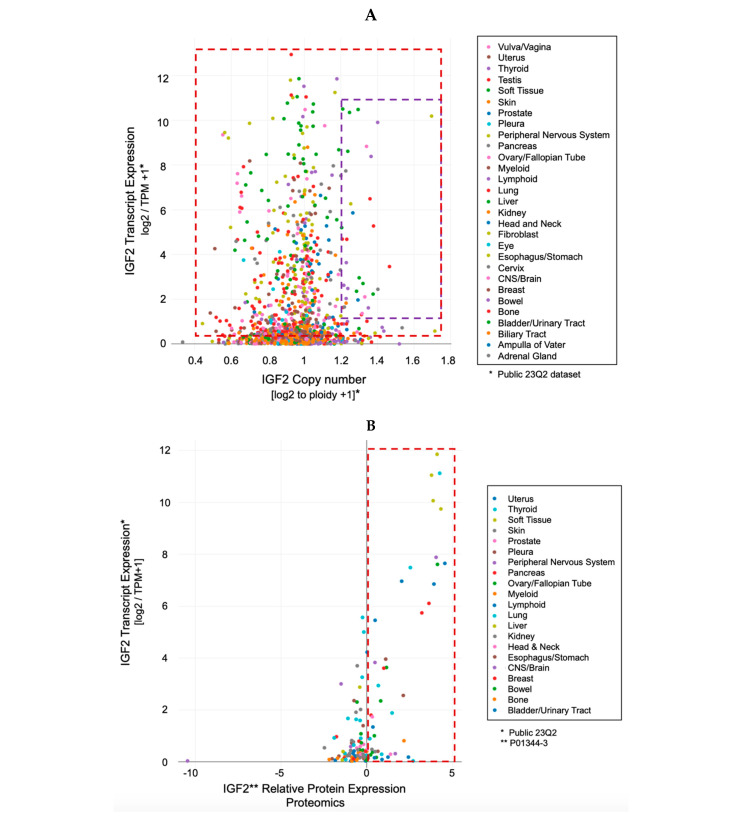

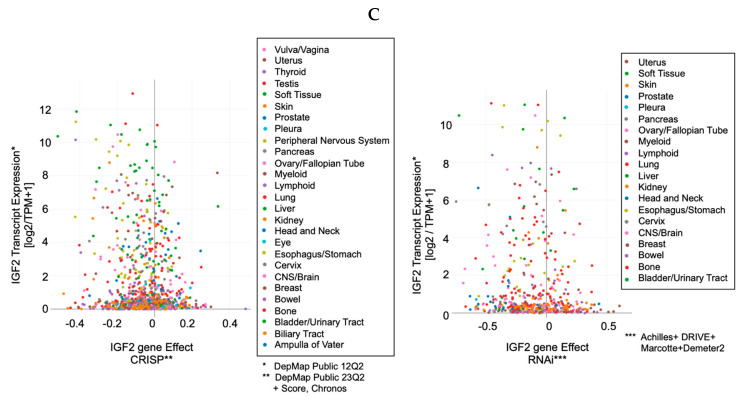
IGF2 (gene, transcript, and protein) expression pattern in patient-derived cancer cells. DepMap-originated expression levels conveyed in Figure are relative to the respective content in normal cells; (**A**) IGF2 transcript expression is a common event in cancer cells (as summarized in the red boxed area) and rarely associates with its gene duplication events (compare red with purple boxed areas); (**B**) IGF-II ligand expression (proteomics) is a common event in cancer cells, even at low transcription expression levels (see red boxed area spanning from the 0 value corresponding to expression levels comparable to normal cells, up to 12 times the IGF2 transcript expression in normal cells); (**C**) comparative effect of IGF2 gene editing (**left**) and/or transcript silencing (**right**) in cancer cell lines. Note the consistent distribution of human cancer cells among those responding to IGF2 gene block (by either CRISP or RNAi) irrespective of the folds of IGF2 transcript native over-expression. The source of the data in Figure 3 is conveyed in the Supplemental Material (SM2).

Indeed, the idea of IGF-II secretion as a rare associated event in cancer has been maintained in the scientific literature till present [29], somehow implying that IGF-II-secreting tumors could be mostly benign in nature and fully surgically treatable. This has motivated a group of authors to name such tumors as “IGF2omas” recalling the rare and surgically removable features of the early reports [5]. However, the cumulative evidence based on expression studies conducted at the histological and cellular level suggests a different scenario than that proposed by clinical reports of its rarer hypoglycemic-associated syndrome. In fact, based on the retrospective analysis of the published literature, which we conveyed herein in Figure 1 and Table 1, it is clear that IGF-II secretion in tumors is a much more common event than generally implied by IGF studies focusing on mechanistic and reductionistic experimental design. Interestingly, IGF-II expression by cancer cells and bioptic tissues from solid malignancies exceeds, by several orders of magnitude, the number of cancers overtly displaying hypoglycemia. Although there is still not sufficient published evidence, increases in hypoxia and CO2 levels with resultant body acidification in cancer patients may also result as a highly associated event with IGF-II secretion in patients diagnosed with a solid tumor. The rational for this predicted association is based on the demonstrated IGF-II expression increase in response to HIF-1 stimuli reported in a variety of experimental cancer models [30]. To further characterize the expression levels and patterns of IGF-II in cancer, we turned to the DepMap expression database, a publicly available tool managed by the Broad Institute [31] (available at https://depmap.org/portal/ accessed on 24 September 2023), and focused on a few key parameters conveyed in Figure 3. This analysis, relative to a number of well-characterized human-derived cancer cell lines, has provided the following results:

1. The IGF-II transcript expression in cancer cells exceeds the expression of normal cells and tissues by a range of 0.1- to 12-fold (Figure 3A–C);
2. The IGF-II transcript (mRNA) expression is not commonly associated with gene duplication events (Figure 3A);
3. The IGF-II protein expression in human-derived cancer cells exceeds normal cells/tissues by 0.1- to 5-fold (Figure 3B);
4. IGF-II gene editing and or transcript silencing negatively affects ~60–65% of cancer cells (Figure 3C).

As for IGF2 expression and its correlation to solid cancer, despite the established association of IGF2 transcript and ligand (IGF-II) expression in a wider spectrum of solid tumors (summarized in Table 1), a few studies have specifically looked at the cause–effect between IGF-II overexpression and malignant switch. Two seminal studies addressing this point are discussed in the following. The first, authored by Rogler et al. and conducted in a IGF2 transgenic mice model [14], observed development of a broad spectrum of solid malignancies (3.25-fold higher than normal control animals), with resulting transgenic mice bearing an IGF2 transgene construct able to drive 20- to 30-times-higher plasma levels than control animals. Interestingly, the study shows that in these mice, hypoglycemia increased proportionally with the increase in the circulating IGF-II levels. In particular, in animals with up to 20 times the mean levels of circulating IGF-II, the measured glycemic levels were still in the normal range despite hypoglycemia being more frequent with aging. On the other hand, all IGF2 transgenic mice displaying more than 30 times the IGF-II levels compared to non-transgenic control animals did constantly display reduced blood glucose levels and symptoms of hypoglycemia. This particular finding implies that increased level of IGF2 transcript expression and consequent IGF-II ligand secretion might affect a larger number of solid malignancies before setting or even in the absence of underlying hypoglycemic symptoms. This is also consistent with the retrospective literature findings conveyed herein (Table 1 and Figure 1) supporting the idea that IGF2 transcript or IGF-II protein expression is a broader event in cancer compared to the established but rarer paraneoplastic hypoglycemic symptoms linked to IGF-II’s non-suppressible insulin-like activity (NSILA) [1], also referred as non-islet-cell tumor hypoglycemia (NICTH) [8] and extra-pancreatic tumor hypoglycemia (EPTH) [32]. A feasible explanation for such discrepancy between actual expression and systemic symptoms occurrence stands on the levels of circulating/systemic IGF-II required to cause hypoglycemic symptoms (able to drive blood glucose below 60 mg/dL and trigger the physiologic compensatory glycolytic response from insulin-counteracting hormones). Indeed, these compensatory mechanisms, by releasing liver-stored glucose, may hinder the hypoglycemic symptoms, along with the actual presence of IGF-II secretion by the tumor, for a very long time. The partial display of hypoglycemic symptoms among patients with IGF-II-secreting tumors reported in the literature is compliant with the possibility that the tissue concentration of IGF-II required to sustain autocrine tumorigenic signals may be several orders lower compared to those needed to trigger generalized hypoglycemia.

A second seminal study clearly linking (a) endogenous tissue focal expression and cancer-secretion of IGF-II (b) with the tumor malignant switch was published by Christofori et al. [15] using an invaluable genetic mice model initially developed by Hanahan et al. [33,34] that recapitulated the stage progression features and requirements of pancreatic carcinoma. In particular, this study provides the first direct evidence of IGF-II’s role as a stage-specific component in the tumor angiogenic switch, the checkpoint at which a growing tumor acquires the capability to make its own blood vessels and acquire independent tridimensional growth features as typically observed during malignant transformation.

## 4. The Role of IGF-II in Cancer Is Not Alternative to IGF-I

Traditionally, IGF-I and IGF-II have been considered almost to be interchangeable and/or redundant ligands triggering the oncogenic effects of the IGF-IR. Nonetheless, unlike IGF-II, IGF-I is not commonly found to be over-expressed or secreted by cancer cells and it has been found to be negligibly associated with NICTH (summarized in Figure 1 and Table 1). Indeed, there are a plethora of studies involving IGF-I in cancer. The current lines of evidence supporting its role can be conveyed in (a) epidemiologic studies associating relatively high levels of circulating IGF-I to increased incidence of prostate, breast, and other cancers [35,36,37,38], and (b) other studies in vitro with human tumor cells implicating IGF-I in growth, survival, migration, and metastatic behavior upon activation of the expressed IGF-IR [39,40,41], as well as resistance to chemotherapeutic and radiation therapies [42].

Physiologically, IGF-I levels in all mammalian species including humans are known to peak during the pubertal phase and slowly decrease throughout lifetime in response to GH, which shares a similar age-related trend [43]. This general concentration decreasing pattern is not different in that group of patients with increased cancer risk, despite such (relative increase in) circulating IGF-I amounts being significantly lower compared to the same subject during pubertal age. In other words, there is no dose–response correspondence between absolute IGF-I levels in blood and cancer risk given the very low prevalence of cancer in the pubertal population.

Noteworthy, in the epidemiologic studies associating higher levels of IGF-I to increased cancer risk, no specific attention has been given to the cellular source or cancer tissue component responsible for IGF-I production. Additionally, while epidemiology has suggested a link between high IGF-I blood levels and increased cancer risk, a cancer-protective role of low IGF-I dose exposures, such as in IGF-I treated subjects affected by Laron syndrome (a genetic form of IGF-1 deficiency), has been more difficult to demonstrate given the fact that these subjects have cancer risk comparable to those exposed to higher IGF-I doses [reviewed by Werner and Laron [44]]. Interestingly, updated FDA recommendations for rhIGF-I usage in IGF-I deficiency conditions warn about increased occurrence of neoplasia, especially when used at higher dosages, including some rare malignancies not typically observed in children. This is in line with the widely described effects of supra-physiological levels of IGF-I stimulation reported in vitro [20].

How, therefore, can these epidemiologic studies associating increased IGF-I levels to increased cancer risk fit with its negligible presence among the paraneoplastic hypoglycemic cases reported in the literature? A potential explanation can be found in the contextual and stage-related expression pattern of IGF1 versus IGF2 at the focal tissue level (tumor microenvironment). In fact, with the sole exception of leukemia, where IGF-I production and secretion is observed more frequently than IGF-II in cancer cells [45], seminal studies on the source of IGFs in cancer cells and bioptic tissues obtained from solid cancers have found IGF-I to be expressed and secreted mostly, if not exclusively, by the stromal component (fibroblast and other stromal cells) [46,47], with its high-affinity RTK receptor (IGF-IR) being variably expressed in both stromal and cancer cells [48]. This proves that IGF-I acts as a paracrine factor in these cancer types and that cancer stroma is a source of IGF-I in these patients. Indeed, it is feasible that cancer stroma may be a major underscored source of circulating IGF-I in those patients with parallel increased cancer risk and that such pools of IGF-I do not correlate with the physiological source of circulating IGF-I, which is mostly produced in the liver and may not change significantly during malignancies affecting other organs or body districts. On the other hand, IGF-II has been found to be expressed and secreted both in cancer cells and in the stromal tissue in the same cancer studies, therefore establishing both paracrine and autocrine stimuli [47]. However, as for the type of IGF-II signal provided in this context, it is worth mentioning that the predominant IGF-II form secreted by cancer cells is its high-molecular weight (big)-IGF-II form which escapes IGFBP-3 and SpI2-6 (IGF2R) binding [49,50] which can biologically differentiate the IGF-II paracrine versus autocrine signal.

As a result, the type of broader evidence currently available to support IGF-I’s role in cancer, suggesting foreseeable advantages in IGF-I targetability compared to the single block of big-IGF-II in cancer, are highly debatable unless and until this is differently demonstrated using appropriate experimental design (namely with selective IGF-I and IGF-II ligands block and using positional biology multi-plex, or better, multi-omic methods to pinpoint the exact cellular source of protein expression in the cancer tissue context). This concept is even more actual on the base of the differential effects of these ligands in terms of malignant switch, as further discussed in the next chapter.

Consistent with the concept of a differential effect of IGFs in cancer, increased IGF-II bioavailability in the tumor microenvironment is also provided by reduced expression of its high affinity scavenger receptor SpI2-6 [12], formerly referred as IGF2 receptor, secondary to its loss of heterozygosity [51,52,53]. Indeed, SpI2-6/IGF2R tumor suppressor functions have been widely demonstrated to be linked to its ability to sequestrate and degrade IGF-II through direct cell internalization [10,54], while this does not apply to IGF-I, which displays negligible binding to SpI2-6/IGF2R at physiological concentrations [55,56]. On the other hand, while locally expressed IGFBPs do bind both IGF-I and IGF-II (7.5 KDa), big-IGF-II variants can escape such binding and exert biological advantages [50].

**Figure 4:**
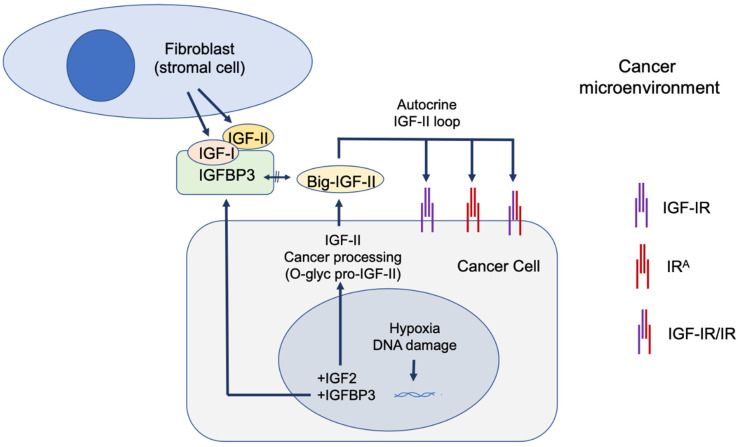
Role of IGFBP-3 in differential IGFs bioavailability in cancer microenvironment. Under hypoxic conditions, IGF2 and IGFBP-3 are upregulated at the transcriptional level and consequently over-expressed at the protein level. In cancer cells, the IGF2 transcript undergoes defective processing, leading to its high molecular variant (big-IGF-II) which is secreted, along with IGFBP3, in the cancer microenvironment. The refractory binding of big-IGF-II with IGFBP-3 favors the selective sequestration of IGF-I and IGF-II secreted by the cancer stromal component [46,68], while big-IGF-II is able to effectively stimulate autocrine parallel signals via the IGF-IR, the IR^A^, and the IGF-IR/IR hybrid variant.

In this regard, IGF-I bioavailability in cancer can be further reduced via IGFBP-3 upregulation, which is triggered by (wild-type) TP53 activation induced via DNA damage and/or hypoxia [57]. Hypoxia also upregulates IGF2 transcription via HIF-1 [58]. This parallel increase in IGF2 transcription, coupled with defective cancer processing, generates the known high-molecular IGF-II pro-hormone variants [11], which are refractive to IGFBP-3 [49,50] (and SpI2-6/IGF2R) binding [50] but not to the IGF-II RTKs (IGF-IR and IR^A^) which are efficiently activated [11,50]. This contextual increase in big-IGF-II and IGFBP-3 in the extracellular microenvironment can ultimately decrease IGF-I bioavailability [59,60] and favor the big-IGF-II autocrine tumorigenic signal and effects. This scenario is likely to play a distinctive role at the transition between benign and malignant growth [15] when the urge for tridimensional growth in the absence of an established vascular network in the growing tissue triggers inner mass hypoxia towards favoring an angiogenic switch. Under these circumstances, based on the above bioavailability scenario, the big-IGF-II autocrine growth stimuli may prevail over the combined IGFs paracrine stimuli. These contextual mechanisms are graphically conveyed in Figure 4. Interestingly, EGFR overexpression also induces IGFBP-3 in cancer cell lines [61], supporting the idea that EGFR and the IGF-II autocrine signals might act synergistically in a variety of solid cancers. It is worth mentioning that such contextual circuitry fits with early-stage tumorigenic phases where TP53 function is maintained. As for those advanced cancers (more than 50%) with loss of function of TP53, this condition has been shown to further trigger IGF2 transcription [18] and further consolidate the ability of a cancer cell to maintain its malignant features. Although the genetic and epigenetic mechanisms underlying IGF2 expression in cancer have been reviewed elsewhere [[28], ibidem] and are not the subject of the present perspective, we included this mechanism as an example of the role of TP53 in the regulation of IGFBP-3, which is directly involved in the high-affinity binding of mature IGF-I and IGF-II but not of cancer-secreted big-IGF-II.

Other factors have been shown to play a mandatory role in IGF-I and IGF-II biosynthesis, such as GRP94 [[62,63], reviewed in [64]]. The relevance of this chaperone protein towards sustaining paracrine and autocrine loops is also suggested by its increased expression in cancer [65,66]. Since GRP94 exerts its maturation-/secretion-promoting activity on IGFs by physically associating to its pro-hormones [62,63], it will be interesting to clarify its specific role towards the production/secretion of big-IGF-II variants given their ability to escape IGFBP proteins’ high-affinity binding. In terms of bioavailability at the microenvironmental level, it is reasonable to think that anytime IGF-I levels potentially escape sequestration/neutralization by extracellular IGFBPs in the cancer microenvironment (e.g., by increased local cleavage of IGFBPs) [67], its signal may provide a further advantage towards cancer cells’ viability and serum independence. Nonetheless, the exact biological impact of IGF-I towards the acquisition and maintenance of malignant features has not yet been demonstrated in vivo, unlike IGF-II [15]. Altogether, the published evidence discussed above further supports differential roles between IGF-I and IGF-II in cancer. Although it has been shown that the IGF-I signal seems to be provided mostly by the cancer-surrounding stromal component [46,47], or what we call the cancer microenvironment, it will be important to evaluate the contribution of stromal IGFs in terms of function and potential synergistic effect with that provided by the big-IGF-II autocrine loop throughout the tumorigenic process. A feasible scenario of this dynamic landscape and individual contribution, in tight relationship with the underlying contextual receptorial system, is provided in Figure 4.

## 5. The IGF-II Cancer Driving Signal Mediating Receptors: An Unexploited Combinatorial Landscape

The IGF-I signal in cancer cells relies on the expression of both the IGF-IR and the IGF1R/IR hybrid. According to published evidence, the IGF-II ligand production/secretion and autocrine signal, linked to the cancer angiogenic/malignant switch [15], can overcome the cancer cell dependence on the IGF-I paracrine signal in more advanced stages. In particular, at this transition checkpoint, the cancer cell acquires the ability to secrete big-IGF-II and establish its autocrine stimulatory loop mediated via diversified signals that big-IGF-II exerts via the IGF-IR and the Insulin receptor fetal variant (IR^A^) [69].

At the cellular level, IGF-I, at the concentrations observed in vivo, is able to activate both its receptor (IGF-IR) as well as the hybrid receptor made by the combination of the IGF-IR alpha-beta component with the homologous hetero-dimer of the Insulin receptor [25,70,71,72,73]. In the case of cancer-secreted (autocrine) IGF-II, even at lower tissue concentrations than those found in the blood of patients with paraneoplastic hypoglycemia, is able to activate the IGF-IR, the fetal IR isoform (IR^A^) which is over-expressed in cancers [16,48], as well as the IGF-IR/IR^A^ hybrids [25].

Therefore, the old view that the IGF-I receptor (IGF-IR) would exclusively mediate growth, proliferative, and anti-apoptotic signals from IGF-I and IGF-II while the Insulin receptor (IR) would mediate metabolic actions in response to insulin is obsolete and does not reflect the actual biology of IGFs in cancer, despite being perpetuated till recently. Additionally, even in the presence of a partially redundant number of co-targeted intra-cellular molecules, the evidence of distinctive intracellular targeting abilities mediated by the two receptors (IGF-IR and IR^A^) upon activation by the same autocrine ligand (IGF-II) (as for the autocrine IGF-II/IR^A^-mediated degradation rescue of EphB4 [19,74]) or the different targeting ability of individual Insulin/IGFs through same receptor (IR^A^) [25,75] has been increasingly demonstrated. Furthermore, a number of published findings have identified Insulin/IGF-ligand-dependent and hetero-dimeric receptor tyrosine kinase type-dependent signals and underlying differentially regulated targets [25,71], supporting the view that a number of distinctive signals are indeed generated in the same cell according to the contextual ligands and receptors co-expression [16,23].

**Figure 5:**
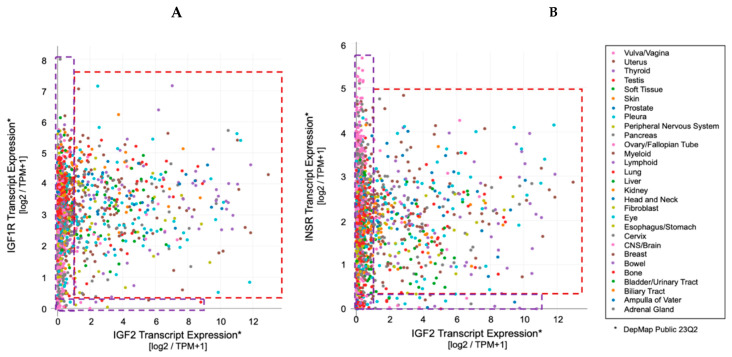
Relative RNA transcript expression of IGF-II transducing receptor tyrosine kinases in cancer cell lines. (**A**) Relative expression of IGF1R versus IGF2; (**B**) relative expression of Insulin receptor versus IGF2. Red boxed areas include those lines with relative expressions >1 fold, both in ligand and receptor, compared to normal and a group of tumoral cells. Purple boxed areas correspond to those tumoral cells with relatively normal levels and isolated elevated levels of either IGF2 or its RTK signal-transducing receptor transcripts. The source of the data used for Figure 5 is conveyed in the Supplemental Material (SM2).

Even at levels of secreted IGF-II comparable to those observed in a subset of tumoral cells, the over-expression in IGF-II signal-transducing RTKs (namely IGF-IR and Insulin receptor fetal variant, up to six times the normal levels as shown in Figure 3) allows cancer cells to effectively respond to the secreted ligand signal in addition to the biological advantages provided by the cancer-specific IGF-II-secreted variant discussed herein and elsewhere [6,76]. Noteworthy, the lack of isoform-specific information in the currently available proteo-transcriptomic data sets constitutes a significant limitation. In fact, in the context of the IGF system, this affects both the quantification of cancer-specific IGF-II (o-glycosylated IGF-II pro-hormone) as well as the insulin receptor fetal isoform variant (IR^A^) in patient-derived cancer cells. In particular, the level and relative quantification of cancer-specific (big)IGF-II can be made possible upon long-read transcript sequencing scouting for the retention of the exon regions coding for the IGF2 D and E domains bearing the glycosylation sites responsible for the glyco-moiety present in its high molecular variants [9]. Alternatively, the same type of information for both IR and IGF-II isoform variants could be drawn upon specific identification of a proteomic peptide corresponding to the alternatively spliced/exon retained region in a proteomic dataset, similar to what has recently been achieved in an IGF canine NICTH model [77]. Until such large sets of intron-inclusion/retention transcriptomic data and/or isoform-specific proteomic data are made available on the same established (patient-derived) cancer cell lines, we can only assume, with an high level of confidence, that the IGF2 cancer-specific isoform variant is present at variable levels in the available datasets, with the highest probability in those cells with higher IGF2 relative transcript expression and with a growing correlation from the transcript to the protein level. Overall, the available transcript co-expression data for IGF2 and its RTK-transducing receptors (IR^A^ and IGF-IR) suggest the following:

(A) The IGF-II signal-transducing RTKs (IR^A^ and IGF-IR) are variably overexpressed from 0.1- to 6-fold in patient-derived cancer cell lines (Figure 5A and B, respectively);
(B) The IGF2 transcript is over-expressed in the same cell lines from 0.1- to 12-fold (Figure 5A,B)

As mentioned earlier, and as further strengthened by the data conveyed in Figure 5 below, a potential yet under-estimated implication of the widespread co-expression of both IR and IGF-IR along with IGF-II in cancer cell lines stands on the combinatorial and yet poorly studied effects of their hybrid receptors. In fact, hybrid receptors made by the IGF-IR holo-dimer with the IR, irrespective of the underlying isoform (IR^A/B^), have been reported to be responsive to IGF-I and IGF-II and insulin [78], which, under the contextual co-expression circumstances found in the majority of cancers cited in the present perspective (favoring big-IGF-II over IGF-I in vivo), makes the big-IGF-II/HR an underscored ligand/receptor system in cancer, along with other yet undetermined potential IR/non-IGF-IR RTK hybrids (similarly to the IGF-IR/ErbB hybrids), the presence which is as yet unaccounted for, along with their impact at the biological and therapeutic strategies level.

## 6. What Makes IGF-II Secretion in Solid Tumors a Hallmark of Malignancy beyond Its Renown Para-Neoplastic Association?

The major features pointing at cancer-secreted IGF-II as the major and widely expressed IGF factor in solid cancer can be summarized in the following established pieces of evidence, potentially affecting present and future pathology screening and therapeutic targeting strategies (further discussed in Section 7):

(A) Cancer tissues, irrespective of their embryological tissue of origin (spanning from sarcomas to carcinomas), express a wide-spanning number of IGF-I receptors (IGF-IR), along with a tumorigenic stage-specific (from benign to malignant) increase in fetal insulin receptor isoform variant (IR^A^). Under such circumstances, cancer cells and tissues will display a variable amount of homogeneous receptors (individual IR and IGF1R) along with an increasing amount of hybrid IGF1R-IR receptors (HRs) directly depending on the increase in the expressed IGF1R (the higher the IGF-IR expression, the higher the amount of HRs) [25].
(B) With the only exception of pancreatic beta-cell benign tumors (insulinomas), which produce an excess of insulin, the vast majority of solid malignancies express IGF-II in higher molecular variants (O-Glycosylated pro-hormone peptides with MW spanning between 15 and 27 KDa [7,74]) which are resistant to extracellular binding and sequestration by physiological IGF binding/scavenging factors, namely IGFBP3 and SpI2-6 [12], improperly referred as IGF-II “receptor” (IGF2R). IGF-II production/secretion has also been shown to be provided by cancer-associated fibroblasts [68], although there is still no evidence that this type of IGF-II belongs to an high-molecular-weight variant. As conveyed in Figure 1 and Table 1, it is worth noting that isolated IGF-I over-expression in cancer cell is a minor event compared to IGF-II over-expression, and it is mostly restricted to its stromal component, supporting its paracrine functions (Figure 4).
(C) IGF-II over-production in cancer is currently demonstrated in the clinical setting in the presence of a diagnosed cancer patient displaying variable glycemic levels, spanning from normal to sub-normal, along with recurrent episodes of hypoglycemic symptoms fully reversible via surgical removal or ablation of the underlying cancer tissue. In these patients, the measured blood IGF-II levels (by ELISA) span from overtly supraphysiologic levels to apparently normal ranges but with a reduced or inverted ratio between IGF-II and IGF-I (normally <10). In all other malignancies not displaying such findings, IGF-II expression could be detected via both traditional histopathologic means in the clinical setting or more accurately (not frequently adopted in the clinical settings) via molecular techniques (namely qRT-PCR and NGS-RNAseq). The inclusion of IGF-II among the high-throughput targets of positional tissue expression panels able to localize and measure specific gene expression patterns within cellular components throughout bioptic tissues will provide a confirmatory tool for both the relative and absolute quantification of the above-cited IGF factors, along with hundreds of other known cancer-driving gene products.

**Figure 6:**
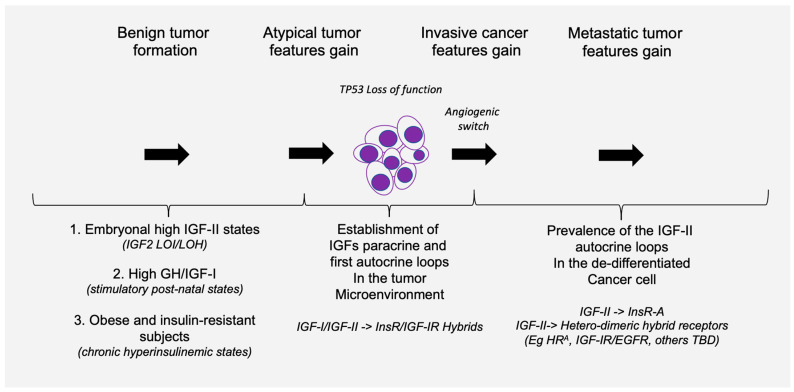
Checkpoint-driven tumorigenic progression model for the role of cancer-secreted (big)IGF-II. A set of clinical, pathological, genetic, cellular, and biomolecular events established in the literature have been conveyed at specific tumorigenic checkpoints, providing the displayed workflow for the stage-associated function(s) of cancer-secreted IGF-II, supporting a specific role for IGF-II at the malignant transition checkpoint in solid cancers.

The factors affecting IGFs bioavailability at the level of cancer micro-environment are conveyed in Table 2. An overview of the working hypothesis for the contextual IGFs and their receptors role in tumor progression conveyed in the present work is graphically summarized in Figure 6.

## 7. Is IGF-II-Secreting Tumor (IGF-IIsT) a Biologically Sounder Acronym for the Role of IGF-II in Cancer Biology?

Previous authors have used the terms Non-Suppressible Insulin-Like Activity (NSILA) [1], Non-Islet Cell Tumor Hypoglycemia (NICTH) [8], and Extra-Pancreatic Tumor Hypoglycemia (EPTH) [32] for the paraneoplastic syndrome that has been associated with those tumors secreting high levels of IGF-II, which, upon release into the bloodstream, determines systemic insulin-like hypoglycemic effects [85,86,87]. The acronym of IGF2omas has previously been proposed for such tumors [5]. This acronym, on the one hand, reflects the established association of this paraneoplastic syndrome to the exclusive production of IGF-II while differentiating them from those associated with insulin secretion (by definition restricted to rare pancreatic insulinomas). On the other hand, the term differentiates this group of tumors from those associated with secondary IGF-I secretion despite the reported studies that have either displayed or omitted co-secretion of IGF-II (Table 1). As discussed herein, IGF-II-associated paraneoplastic condition is a much rarer occurrence compared to the number of IGF-II-secreting malignancies that do not (yet) display such symptoms [88]. Despite the fact that, to date, no large-scale studies have yet been produced to determine the rate of solid cancers expressing/secreting IGF-II, the DepMap analysis from patient-derived cancer cells conveyed in Figure 3B suggests that such a number may exceed 65% percent. Based on the wider and underscored occurrence of IGF-II secretion among solid malignancies, independently from the circulating levels clinically associated with hypoglycemia, we find it to be more appropriate to classify tumors as either IGF-II-secreting tumors (IGF-IIsT) or non-IGF-II-secreting tumors (non-IGF-IIsT). Specifically, for IGF-IIsT, we refer to all cancers positive for IGF-II secretion (usually displaying various degrees of dedifferentiation associated with a high IR^A^/IR^B^ expression ratio), which confers malignant and aggressive tumor behavior. IGF-IIsT, therefore, includes the smaller group formerly defined as IGF2omas and further extends to all IGF-II-bearing autocrine loop-positive cancers, as confirmed via (a) histopathological or other protein-positional pathology detection/imaging methods, (b) transcript (RNAseq) or protein (mass-spectrometry-based) panels in patient-derived circulating tumor cells (CTCs), or (c) IGF-II-neutralizing antibody-inhibited cultural growth of patient primary cells or CTC cultures. In particular, the latter two methods (in circulating tumor cells) provide ex vivo testable parameters attainable as part of a liquid biopsy. These approaches can be several magnitudes more sensitive compared to the cancer patient IGF-I/IGF-II high blood ratio currently reported as the discriminating metabolic hallmark for paraneoplastic hypoglycemia-associated cancers [28]. In this context, the proposed parameters for inclusion of a solid tumor under the proposed IGF-IIsT extended group include previously described tumors with the following features:

(a) Any tumor mass, independent of its size or staging, characterized by inner mass hypoxic conditions (e.g., by CT/PET-FDG) and underlying angiogenic switch, preceding any other histopathological feature coupled with an inversion of the circulating detectable IGF-I/IGF-II ratio (with IGF-II > IGF-I);
(b) Any histopathology or molecular biology report of a solid tumor bioptic specimen displaying co-expression of pre-pro-IGF-II (associated with its cancer-secreted high-molecular weight variant) and Insulin receptor fetal isoform (IR^A^) transcripts;
(c) Any undetected metastatic foci in a previously diagnosed patient or in an apparently normal subject with familiarity for solid cancer in which a circulating tumor cell (CTC) can be isolated and analyzed using available high-sensitivity single-cell applicable methodologies (dPCR).

From a biological standpoint, it is worth noting that, given the ubiquitous expression of the Insulin receptor and its fetal variant’s increased levels in cancer cells, coupled with the variable expression patterns of the IGF-IR, the simple IGF-II transcript detection via available molecular detection methods identifies, bona fide, an autocrine IGF-II loop and, therefore, an IGF-II secreting tumor (IGF-IIsT).

The cumulative features conveyed in the IGF-IIsT group definition, we believe, may offer a biologically sounder context for both classification and future personalized molecular targeting strategies pointing at cancer-secreted IGF-II and its malignant-switch-specific intracellular signal as a widely evidence-based target over any of its individual receptors.

## 8. Conclusions and Perspectives

Published work on the Insulin/IGF ligands and receptorial system in cancer provides an emerging landscape with a distinctive role for cancer-secreted IGF-II and its autocrine effects. The latest findings associating IGF-II autocrine loops in cancer to the Over-Expression-by-Degradation Rescue (OEDR) of an IGF-II signal-targeted angiogenic/oncogenic RTK [19] adds an underscored mechanistic tool used by cancer-secreted IGF-II to exert its tumorigenic effects. Our present work is compliant with a differential role and significance of endocrine IGFs compared to those acting in the extracellular environment during tumorigenesis. Such a working hypothesis envisions systemic (bloodstream) circulating IGFs in cancer as an epiphenomenon rather than a causal and/or primary risk factor. Indeed, our interpretation of the reviewed literature supports a view by which circulating IGFs levels are secondary to underlying processes such as cancer-associated inflammation [89] and/or tumor-microenvironment-mediated hormonal/growth factor loops [90,91,92,93], where prolonged, locally enhanced IGFs signals in parenchymal and/or stromal cancer components may underlie the epidemiologically (IGF-I) and/or clinically (hypoglycemia by big-IGF-II) reported ligand-specific systemic spillover effects. Our analysis of the published literature on the role of IGFs with specific regards to solid cancers is compliant with our evidence-based premises, pointing at a differential production source (IGF-I from cancer stroma acting as paracrine factor, and IGF-II from overt cancer cells acting as an autocrine factor, respectively) and pattern (with pro-hormone IGF-II variant being preferred in the cancer-secreted form). This, coupled with the aforementioned increase in the fetal variant of the insulin receptor (isoform A) at the malignant switch checkpoint, along with the synergistic and differential role provided by the IGF-IR variable expression in tumorigenesis towards cell growth and survival, provides a contextual framework on which to modulate future molecular and therapeutic interventions. Altogether, analysis of the published evidence and publicly available expression dataset conveyed herein further strengthens an actionable role for cancer-secreted (big)IGF-II, with a wider impact compared to its sole detection in the clinically associated paraneoplastic hypoglycemic syndrome.

## Figures and Tables

**Table 1:** Literature analyses of cancer case reports involving IGF-I and IGF-II in relation to cancer-associated hypoglycemia.

	Cancer Associated Hypoglycemia		Reports of Secreted Autocrine/Paracrine Growth Factor		Reporting Elevated Plasma Growth Factor		Reports of Elevated *IGF* Gene Transcripts Level in Underlying Tumor		Cancer Case Report (1972)		Hypoglycemia Case Reports
	IGF-I	IGF-II	IGF-I	IGF-II	IGF-I	IGF-II	IGF-1	IGF-2	IGF-I	IGF-II	
Cancer associated hypoglycemia	Total cancer associated hypoglycemia cases = 1949		18	171	66	24	1	2	1690	1690	
Protein expressing/Secreted IGF	18	171	1644 *	3830	301	201	312	136	136	1657	
Reporting elevated plasma IGF	66	24	322	201	893	892	172	16	22	64	
Cancer (case report)	1656	1656	980	1644	22	48	5	2	623,826	623,826	
											7616

* Mostly associated to stromal component secretion.

**Table 2:** Features affecting IGFs availability and oncogenic effects.

*Feature*	*Ligand*	*Effect(s)*	Efficiency	*Biological/Clinical context*	*Reference(s)*
*Binding to* *IGF-IR*	IGF-I	cell growth, pro-mitotic, anti-apoptotic	+++	Extracellular/Tumor microenvironment	Li et al., 1997 [79] Peruzzi et al., 1999 [80]
	IGF-II	cell growth, pro-mitotic, anti-apoptotic	++		Potalitsyn et al., 2023 [50]
	Big-IGF-II	cell growth, anti-apoptotic pro-tumorigenic	++		Potalitsyn et al., 2023 [50]
Binding to IR^A^ (*)	IGF-I	Negligible at physiological levels	−/+	Extracellular/Tumor microenvironment	Frasca et al., 1999, Sciacca et al., 1999 [16,48]
	IGF-II	IGF-I-like (**) plus pro-angiogenic and pro-invasive	++		Frasca et al., 1999, Morrione et al, 1998, Louvi et al., 1998, [16,23,81]
	Big-IGF-II	IGF-I-like (**) plus events linked to malignant switch	++		Greenhall et al., 2013, Ulanet et al., 2010, Scalia et al., 2019, Potalitsyn et al., 2023 [11,50,69,74]
*Binding to* *SpI2-6/IGF2R*	IGF-I	n/d	−	Extracellular/Tumor microenvironment	Nissley et al., 1984 [55] Bond et al., 2000 [49]
	IGF-II	Overgrowth rescue, IGF-II degradation	+++		Lau et al., 1994 [82], Oka et al., 1985 [10]
	Big-IGF-II	Maintenance of (big)IGF-II Extracellular bioavailability	−/+		Greenhall et al., 2013 [11] Potalitsyn et al., 2023 [50]
*Binding to* *IGFBP-3*	IGF-I	Decrease bioavailability, IGF-independent TBD	+++	Extracellular/Tumor microenvironment	Grimberg et al., 2005, Silha et al., 2006, Takayoka et al., 2007 [57,59,60]
	IGF-II	Decrease bioavailability (***), IGF independent (TBD)	++/+++		Potsalitsyn et al., 2023 [50]
	Big-IGF-II	Maintained bioavailability	−/+		Potsalitsyn et al., 2023 [50]
*Source and effects in Cancer*	IGF-I	Tropic, survival, Pro-tumorigenic (TBD)	Stroma	normal tissue, solid malignancies	Yee et al., 1989, Cullen et al., 1992, [46,47]
	IGF-II	IGF-I-like (**), pro-tumorigenic, immune-evasion	Stroma	solid malignancies	Rogler et al., 1994, [14], Cullen et al., 1992, Yee et al., 1989, [46,47] Belfiore et al., 2023 [83]
	Big-IGF-II	Pro-tumorigenic, events linked to malignant switch	Cancer cell	solid malignancies, paraneoplastic hypoglycemia	Doughaday et al., 1989, Christofori et al., 1994 Dynkevich et al., 2013 [5,6,15]

(*) Feature/effect confirmed in vivo; (**) cell growth, pro-mitotic, anti-apoptotic effects; (***) A colorectal cancer study suggests a cancer-protecting role for decreased IGFBP-3 levels and differentiating/benign-driving role for increased IGF-I bioavailability (Baciuchka et al., 1998) [84].

## Data Availability

Data are contained within the article and supplementary materials.

## Supplementary Materials

The following supporting information can be downloaded at: https://www.mdpi.com/article/10.3390/biomedicines12010040/s1, SM.1: Literature analysis of cancer-associated hypoglycemia case reports in Table 1 and Figure 2. SM.2: DepMap data conveyed in Figure 3 and Figure 5 for the cancer cell lines-based co-expression and/or silenced-gene expression analysis.
